# Cellular senescence in lung cancer immune microenvironment and senotherapeutic opportunities

**DOI:** 10.1016/j.isci.2026.114673

**Published:** 2026-01-12

**Authors:** Zhaolin Xu, Fangfang Liu, Shanshan Huang, Qian Chu

**Affiliations:** 1 Department of Oncology, Tongji Hospital of Tongji Medical College, Huazhong University of Science and Technology, Wuhan 430030, China

**Keywords:** therapeutics, immune system, cancer

## Abstract

Cellular senescence (CS) shapes lung cancer biology by enforcing durable growth arrest while remodeling the tumor immune microenvironment (TIME) through the senescence-associated secretory phenotype (SASP). In non-small cell lung cancer (NSCLC), immune checkpoint inhibitors (ICIs) have improved outcomes, and senescence programs can influence antigen presentation, immune cell recruitment, and adaptive resistance. This review integrates evidence from cell and animal models, patient-derived datasets, and early clinical studies to map senescent cell states across cancer immunoediting and treatment contexts. We summarize senescence phenotypes in tumor and stromal compartments, highlight SASP-driven immune modulation, and discuss senescence-linked biomarkers that may inform prognosis and therapy selection. We also evaluate emerging senotherapeutic strategies—senolytics, prodrug approaches, and SASP-modulating senomorphics—designed to pair senescence induction with selective clearance or reprogramming. Together, these concepts frame a testable “induce, then edit or eliminate” paradigm to improve durability of lung cancer therapies.

## Introduction

Lung cancer is the leading cause of cancer-related morbidity and mortality, responsible for 12.4% of global diagnoses and 18.7% of cancer deaths (GLOBOCAN 2022). Lung cancer is often asymptomatic in its early stages, resulting in most diagnoses occurring at advanced stages. In China, the 5-year survival rate remains modest at 20%–30%, underscoring the urgent need for improved therapeutic strategies. Immune checkpoint inhibitors (ICIs) targeting PD-1/PD-L1 have revolutionized survival in advanced non-small cell lung cancer (NSCLC) and are now first-line treatments. However, immune responses can be influenced by factors within the tumor microenvironment (TME). Cellular senescence (CS) is the irreversible arrest of the cell cycle and is triggered by stressors such as DNA damage and oxidative stress. A key feature of CS is the senescence-associated secretory phenotype (SASP), which releases pro-inflammatory factors.^1^ CS-mediated SASP and cytokine storms impair anti-tumor immunity by altering the TME through mechanisms such as senescence-induced metabolic dysfunction,^2^ immune cell phenotype reprogramming,^3^ and chronic inflammation.^4^ Emerging evidence suggests that CS may compromise these immunotherapeutic gains. A comprehensive understanding of these processes is essential for improving therapeutic outcomes in lung cancer.

## CS

CS refers to a state in which cells irreversibly cease to divide and enter a prolonged growth arrest following exposure to various forms of stress or damage. CS can be classified into replicative and premature senescence, both of which are intricately linked to physiological and pathological processes such as aging, tumorigenesis, and tissue repair.

### Types of CS

CS is generally categorized into two main types: replicative senescence (RS) and premature senescence. RS, also known as telomere-dependent senescence, is a natural physiological process linked to cellular aging. In contrast, premature senescence is triggered by various stressors, such as oncogene activation, genotoxic agents, or oxidative stress. Each type is driven by distinct molecular mechanisms and contributes differently to physiological and pathological processes.

#### RS

RS was first described by Hayflick in 1961, who proposed that cultured cells possess a finite proliferative capacity. With each division, the telomeres of the cells shorten progressively. Upon reaching a critical length, telomeres are recognized as DNA lesions, thereby initiating the DNA damage response (DDR). In a now widely accepted canonical model, established in human lung fibroblasts, this DDR activates critical kinases such as ataxia telangiectasia mutated (ATM) and ATM and Rad3-related (ATR) kinases. The tumor suppressor p53 acts as a key cell cycle regulator that senses various intracellular and environmental stresses, including DNA damage, oxidative stress, and telomere attrition. Upon activation, p53 induces the expression of downstream targets such as p21^CIP1^. The cyclin-dependent kinase inhibitor p21^CIP1^ inhibits CDK2 and CDK4, thereby preventing G1/S phase progression and enforcing cell-cycle arrest.^5^ Additional regulators such as ADP-ribosylation factor 6 (ARF)^6^ modulate p53 stability and activity. The combined activity of these regulators dictates cellular entry into senescence. The tumor suppressor p16^INK4a^ represents another key regulator. It binds to CDK4/6, blocking their activity and preventing the phosphorylation of the retinoblastoma (Rb) protein. Hypophosphorylated Rb sequesters E2F transcription factors to restrain S-phase entry; when Rb becomes phosphorylated, E2Fs are released and cells enter S phase.^7^

#### Premature senescence

**Figure 1:**
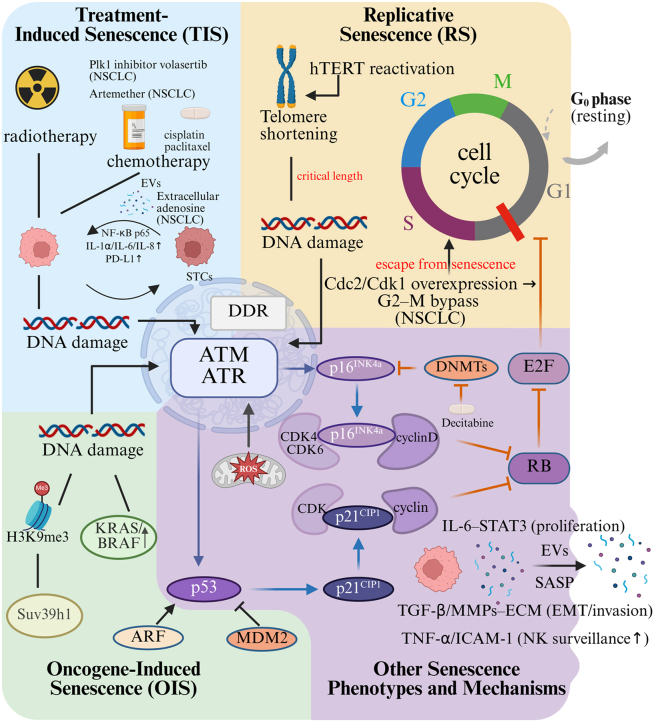
Molecular mechanisms of cellular senescence telomere shortening during replicative senescence, as well as various stresses, cause DNA damage and DDR, which activates ATM/ATR Subsequently, RB phosphorylation is blocked by activating downstream p53/p21 and p16 pathways. Phosphorylated RB will activate E2F and promote the cell cycle progression. Entry routes include treatment-induced senescence (TIS), e.g., cisplatin/paclitaxel; the PLK1 inhibitor volasertib (NSCLC, partly p53-dependent); extracellular adenosine (NSCLC, p53/p21); and artemether (NSCLC, p16-dependent, p53-independent); replicative senescence (RS) driven by telomere shortening, opposed by hTERT; and oncogene-induced senescence (OIS) in KRAS/BRAF contexts via the ARF-p53-MDM2 module. In lung cancer, senescent cells export EVs and cytokines that reprogram tumor cells through IL-6-STAT3 (proliferation/survival), TGF-β/MMP-driven ECM remodeling and EMT (invasion), and cGAS-STING-NF-κB-mediated PD-L1 upregulation (immune evasion), whereas TNF-α/ICAM-1 can enhance NK surveillance. A minority of TIS cells can escape; in NSCLC (TP53-null, CDKN2A-deficient) aberrant Cdc2/Cdk1 enables G2-M bypass and resumed cycling. Dark-blue arrows indicate activation pathways, orange inhibition, and gray direct effects. Created with BioRender.com.

Under specific conditions, including oncogene overactivation, DNA lesions, hypoxia, or metabolic imbalance, cells can enter senescence before critical telomere shortening occurs. This is known as premature senescence. Major subtypes include oncogene-induced senescence (OIS) and therapy-induced senescence (TIS), both serving as tumor-suppressive barriers but with context-dependent impacts on cancer progression, as discussed below. The underlying mechanisms are depicted in Figure 1. OIS, initially characterized in 1997,^8^ represents a form of premature senescence triggered by the overactivation of oncogenes. OIS functions as a tumor-suppressive mechanism by enforcing cell-cycle arrest, thereby preventing uncontrolled proliferation and oncogenic transformation. OIS-associated pathways differ based on the oncogenic driver involved. Ras activation, for instance, drives senescence via coordinated upregulation of p53 and p16^INK4a^. Subsequent pathway activation culminates in G1-phase cell cycle blockade.^9^ OIS exhibits potent tumor-suppressive activity. Suv39h1-mediated H3K9me3 deposition has been shown to enforce senescence in lymphocytes from Eμ-*N*-*Ras* transgenic mouse model, thereby suppressing early lymphomagenesis. This serves as an early barrier in lymphoma development.^10^

TIS, first described by Gewirtz et al., is a cellular response to chemotherapy and radiotherapy characterized by a senescence-like phenotype and cell-cycle arrest.^11^ DNA damage from these therapies activates the DDR, particularly the ATM/ATR and p53/p21^CIP1^ pathways. Elevated reactive oxygen species (ROS) levels further amplify DDR signaling, reinforcing senescence. TIS can be induced by chemotherapeutic mechanisms: (1) topoisomerase inhibitors (e.g., doxorubicin and etoposide)^12^; (2) platinum agents (e.g., cisplatin)^13^; and (3) microtubule inhibitors (e.g., paclitaxel).^14^ While chemotherapy often induces senescence, its primary action in most cancers is apoptosis, achieved through the activation of death receptors or mitochondrial cytochrome *c* release. Radiotherapy similarly activates ATM/ATR signaling, promoting senescence. Consistently, irradiation activates ATM/ATR-p53 signaling and induces senescence in NSCLC models.^15^ Paradoxically, residual senescent tumor cells (STCs) may acquire pro-tumorigenic properties. Zhang et al. demonstrated, in colorectal cancer (CRC) patient cohorts, irinotecan (CPT-11)/ionizing radiation (IR)-induced HCT116/RKO models, and BALB/c nude xenografts, that CRC STCs secrete SERPINE1-rich extracellular vesicles (EVs), which activate nuclear factor κB (NF-κB) p65 and drive tumor progression.^16^ This mechanism illustrates how STC-EVS can be pro-tumorigenic and is likely generalizable beyond CRC.

Oxidative stress-induced senescence is commonly triggered by mitochondrial dysfunction, hyperglycemia, and metabolic dysregulation.^17^ This process is mediated by excessive ROS production and impaired antioxidant defense systems.^18^ ROS promotes senescence via protein oxidation, DNA damage, and DDR activation.^19^ Epigenetic changes, including DNA methylation, histone and RNA modifications, and non-coding RNAs, modulate senescence. To illustrate, the DNA methyltransferase inhibitor decitabine induces CpG demethylation in the p16 promoter, promoting p16 expression and driving senescence in human mesenchymal stem cells (hMSCs).^20^ Senescent cells exert non-cell-autonomous effects via paracrine factors. Notably, SASP signals can reprogram neighboring normal cells toward senescence in culture and in human/mouse OIS systems.^21^ Moreover, in aging-mouse models, bone marrow macrophages (BMM)-derived EVs convey senescence cues to neighboring cells; their microRNA cargo downregulates PPARγ (peroxisome proliferator-activated receptorγ), thereby amplifying paracrine senescence.^22^

#### SASP

SASP is a hallmark of CS, characterized by the secretion of a complex mixture of cytokines, chemokines, growth factors, and proteolytic enzymes. SASP production is a pivotal event in the senescence process. SASP significantly influences the tissue microenvironment by promoting inflammation, accelerating aging, and contributing to age-related pathologies.^23^ Multiple signaling cascades regulate SASP, with the NF-κB pathway playing a central role in its activation.^24^ In irradiated NSCLC cells (A549), radiation-induced senescence (SA-β-gal, p21) is accompanied by increased NF-κB activity and SASP cytokines (interleukin [IL]-1α, IL-6), anchoring this pathway in a lung-cancer context.^25^ The composition of SASP is temporally dynamic and varies depending on the senescence-inducing stimulus, duration, and cell identity. In lung cancer, SASP profiles differ between STCs and other compartments (e.g., cancer-associated fibroblasts [CAFs]), mapping to distinct functional outputs.^26^ To make these cell-type differences explicit, we summarize representative lung-cancer datasets in Table 1.

**Table 1:** SASP composition by cell type in lung cancer and associated outcomes

Cell source	Model and species	Inducer/context	Representative SASP	Tumor effects	Immune effects	Reference
STC (tumor cells)	A549/H1975, human NSCLC (*in vitro*)	IR/TIS	IL-1α, IL-6/IL-8, VEGF, MMPs	EMT/ECM remodeling, migration ↑	PD-L1 ↑	Tesei et al.^25^; Liu et al.^27^; Hwang et al.^28^
Myeloid/TAM	NSCLC patients, mouse models	therapy-adapted SASP	IL-10, TGF-β, CCL2	growth support	MDSC recruitment, CTL suppression	Wang et al.^29^
T cells (TILs/Treg)	human NSCLC TILs; co-culture models	chronic DDR/p38; TME cytokines	CD57/KLRG1↑, p16/p21; PD-1^high^; IL-10/TGF-β (Treg)	–	immunosenescence/exhaustion; Treg-mediated suppression	Zeng et al.^30^; Ikeda et al.^31^
NK cells	intratumoral/peripheral NK in NSCLC	SASP exposure; TGF-β	NKG2D ligands (on targets), ICAM-1; IFN-γ/TNF-α	direct tumor killing (context-dependent)	cytotoxicity ↑ with TNF-α/ICAM-1; suppressed by TGF-β	Sagiv et al.^32^; Ruscetti et al.^33^; Borrelli et al.^34^
CAF (senescence-like)	human lung fibroblasts; KRAS-LUAD mouse	IR/chronic stress	TGF-β, MMPs, CXCL12	growth/metastasis ↑	T cell exclusion	Meng et al.^26^
Endothelium/others	RT-exposed lung vasculature/co-culture	RT/TIS	VEGF, ICAM-1, CXCLs	angiogenesis ↑	immune trafficking changes	Wang et al.^35^; Coppé et al.^36^

Due to its heterogeneous nature, SASP exhibits a dual role in tumor biology. Initially, SASP facilitates immune clearance by recruiting M1 macrophages, Th1 CD4^+^ T cells, and natural killer (NK) cells via chemokines such as CCL2, with IL-1α acting as an upstream alarmin that amplifies SASP and inflammatory signaling.^37^^,^^38^ With prolonged exposure, especially in NSCLC settings, SASP may shift toward tumor promotion by driving epithelial mesenchymal transition [EMT]/extracellular matrix [ECM] remodeling (e.g., IL-6/STAT3 signaling in lung cancer cells; matrix metalloproteinase 1 [MMP1]/transforming growth factor [TGF]-β programs that induce fibroblast senescence and enhance invasion), and by stimulating angiogenesis (senescent fibroblasts upregulate VEGF and increase tumor vascularization).^27^^,^^35^^,^^36^^,^^39^^,^^40^ SASP also fosters immune suppression, for example, by CCL2-CCR2-driven myeloid-derived suppressor cells (MDSCs) recruitment in lung cancer models/patients and by TIS that upregulates PD-L1 on cancer cells.^29^ These effects depend on cell source (STC vs. fibroblast/macrophage), temporal window (acute TIS vs. chronic states relevant to immunoediting), and treatment context (e.g., radiotherapy [RT]/chemotherapy-induced SASP) in NSCLC.^28^

## Role of CS in tumor progression and cancer immunoediting

**Figure 2:**
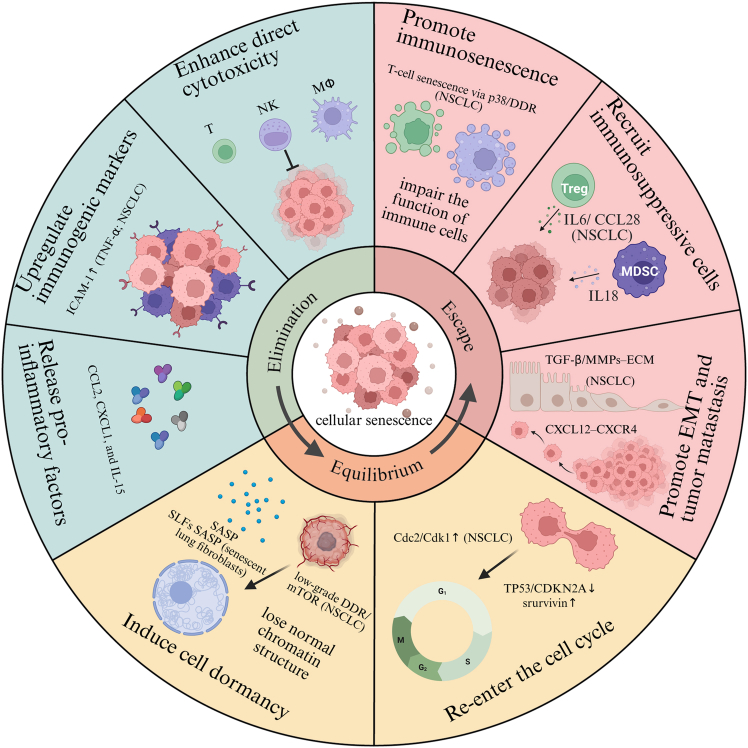
Representation of the role of cellular senescence in different stages of immunoediting (created with BioRender.com)

Understanding the role of CS in the tumor immune microenvironment (TIME) requires acknowledging the intricate interplay between tumor progression and the immune system. The immune system can either suppress or facilitate tumor progression, depending on the context and microenvironmental cues. Dunn et al. conceptualized cancer immunoediting as a triphasic process consisting of elimination, equilibrium, and escape phases.^41^ CS exerts dynamic influence across each phase of immunoediting, modulated by microenvironmental signals and temporal factors. A schematic overview of CS-mediated immunoediting roles is provided in Figure 2.

### Elimination phase

In the elimination phase, antitumor immunity effectively detects and eradicates malignant cells, thereby suppressing tumor progression. STCs enhance immune surveillance by secreting inflammatory mediators that recruit effector immune cells to the TME for targeted clearance. In p53-deficient murine liver carcinoma models, transient reactivation of p53 leads to complete tumor regression. This response is mediated through p53-induced expression of chemokines such as CCL2, which recruit macrophages, neutrophils, and NK cells to the tumor site.^42^ In NSCLC models, RT-induced senescence in A549 increases CCL2/IL-1α and NF-κB activity, facilitating macrophage/Th1/NK recruitment.^25^ STCs upregulate immunogenic markers. In an Nras^G12V^-driven mouse model of hepatocellular carcinoma, senescent hepatocytes show increased expression of major histocompatibility complex (MHC) class II molecules, while upregulation of CD86 enhances CD4^+^ T cell activation. These interactions promote macrophage-mediated clearance of premalignant senescent cells.^43^ Martin et al. demonstrated that persistent STCs secrete immunostimulatory molecules, exhibit increased antigenicity, and remodel their immunopeptidome to enhance immune recognition. These findings were reproduced across multiple human and murine cell lines (e.g., melanoma SKMEL-103 and pancreatic Panc02) and primary fibroblasts, and were corroborated by *in vivo* immunization in C57BL/6 mice.^44^

CS also enhances the cytotoxic function of immune cells. NK cells actively recognize and eliminate senescent cells. Senescent cells overexpress ligands for activating NK receptors such as NKG2D and DNAM-1, facilitating their recognition and elimination by NK cells.^32^ For NSCLC specifically, in KRAS-mutant lung cancer models, NK cell activation dependent on SASP was observed during treatment with MEK and CDK4/6 inhibitors.^33^ TIS induces cross-presentation of the IL-15/IL-15RA complex on tumor cells, promoting NK cell activation and proliferation.^34^ Concurrently, in NSCLC model, TIS upregulates intercellular adhesion molecule-1 (ICAM-1) in A549, enhancing NK cell-mediated cytotoxicity.^33^ These findings suggest that STCs may actively engage NK cell-mediated immune responses.^45^

### Equilibrium phase

When tumor eradication is incomplete during the elimination phase, a dynamic equilibrium is established between residual tumor cells and host immune responses. During this stage, adaptive immunity (primarily CD8^+^ T cells and Th1 cells) actively constrains tumor expansion through cytokine signaling (e.g., interferon [IFN]-γ and tumor necrosis factor alpha [TNF]), which induces senescence in tumor cells by activating p16^INK4a^/Rb pathway and sculpts neoantigen profiles through immunoediting.^46^ This immune pressure maintains tumor dormancy by locking cancer cells in a senescent state, a form of “in-depth dormancy” characterized by irreversible cell-cycle arrest. A defining feature of this phase is the gradual accumulation of senescent cells, which may contribute to tissue dysfunction through aberrant chromatin remodeling. Critically, senescent cells secrete a complex array of factors (SASP) that profoundly shapes the TME recruiting immunosuppressive myeloid cells, especially MDSCs, through pro-inflammatory chemokines such as IL-6, IL-8, and CCL2, thereby establishing an immune-evasive niche^29^; propagating paracrine senescence in neighboring cells via TGF-β-dependent programs, which expands dormancy and selects resistant clones; and directly dampening T cell function through Dickkopf WNT signaling pathway inhibitor 3 (DKK3) and PD-L1.^21^^,^^47^ In lung cancer, these pathways are exemplified by CCL2-CCR2-driven MDSC accumulation in NSCLC patients and mouse models and by TIS upregulating PD-L1 on tumor cells.^48^

Although CS was once considered irreversible, increasing evidence suggests that senescent cells may, under specific conditions, re-enter the cell cycle. Roberson et al. identified subpopulations of TIS cells capable of bypassing growth arrest.^49^ They illustrated that, in the p53-null, p16-deficient human non-small cell H1299 carcinoma cells, aberrant expression of Cdc2/Cdk1 in these cells allows rare TIS cells to bypass G2-M arrest and resume proliferation; analogous escape cells have been observed in resected human lung cancers.^49^ Mechanistically, escape is facilitated by (1) loss of canonical brakes (e.g., TP53/CDKN2A deficiency as in H1299) together with survivin upregulation in the escape cohort, which switches the TIS fate from stable arrest toward re-entry^50^; (2) pathway-level reactivation that sustains survival of drug-tolerant residual disease in lung cancer—most notably YAP (Yes-associated protein)/TEAD (TEA domain transcription factors) signaling in EGFR-mutant NSCLC residual tumors that can regrow upon tyrosine kinase inhibitor (TKI) withdrawal, and mTOR/low-grade DDR rebound that maintains a viable, reversible arrest state.^51^

### Escape phase

In the escape phase, tumor cells evade immune surveillance, leading to uncontrolled proliferation and metastasis. Beyond tumor cells, other components of the TME also acquire senescent features. CS contributes to tumor proliferation and promotes EMT. Senescent human umbilical cord-derived mesenchymal stem cells (s-UCMSCs) significantly enhance the proliferation and migration of breast cancer cells via IL-6/STAT3 signaling.^52^ Human fibroblasts rendered senescent by bleomycin treatment secrete matrix metalloproteinases (MMPs), which in turn promote tumor cell proliferation.^53^ Ultraviolet B (UVB)-induced senescent fibroblasts activate the PI3K/AKT and ERK pathways through ECM components, thereby driving premalignant expansion.^54^ In addition, researchers have demonstrated that SASP derived from senescent fibroblasts induces classical EMT in non-invasive breast cancer cell lines, such as T47D and ZR75.1.^55^ SASP also recruits immunosuppressive cells, including MDSCs and regulatory T cells (Tregs), thereby impairing anti-tumor immunity. Senescent stroma generates MDSC-rich microenvironment via IL-6-dependent SASP in mice. This suppresses CD8^+^ T cells activity and facilitates tumor progression.^56^

Immunosenescence refers to the age-associated decline in immune function and the development of dysfunctional immune responses. SASP contributes to the induction of T cell senescence. Senescent T cells suppress both antigen-independent and allogeneic T cell proliferation, as shown across co-cultures with 012SCC (squamous cell carcinoma of the head and neck), MCF-7 (breast carcinoma), HCT-116 (colorectal carcinoma) and melanoma (MEL-624), thereby impairing adaptive immune responses.^57^ Senescent cytotoxic T lymphocytes (CTLs) exhibit downregulation of key cytolytic molecules such as perforin and granzyme B. Additionally, senescent CD4^+^ T cells secrete pro-inflammatory cytokines such as IL-6 and TNF-α and inhibitory cytokines such as IL-10 and TGF-β.^58^ Senescent macrophages exhibit reduced expression of Mrc1 (CD206), leading to impaired phagocytic capacity.^59^ Senescent neutrophils demonstrate diminished phagocytic activity and chemotaxis.^60^ Furthermore, CS contributes to the acquisition of immunosuppressive phenotypes in MDSCs, enhancing radioresistance in pancreatic cancer.^61^ In lung cancer specifically, immunosenescent remodeling has been observed in NSCLC: tumor and blood compartments harbor enriched senescence-associated CD8^+^ T cell subsets (e.g., CD57^+^ and KLRG1^+^ populations),^30^ and TIS upregulates PD-L1 on cancer cells, constraining T cell effector function.^28^

In summary, the cancer immunoediting model clarifies how CS shapes the tumor-immune interplay across elimination, equilibrium, and escape. In lung cancer, appreciating this dual role—immune surveillance early versus immune evasion and progression when chronic—can guide context-specific interventions (e.g., senescence-targeted strategies and rational combinations with immunotherapy) to improve clinical outcomes.

## Influence of CS on tumor immunity in lung cancer

**Figure 3:**
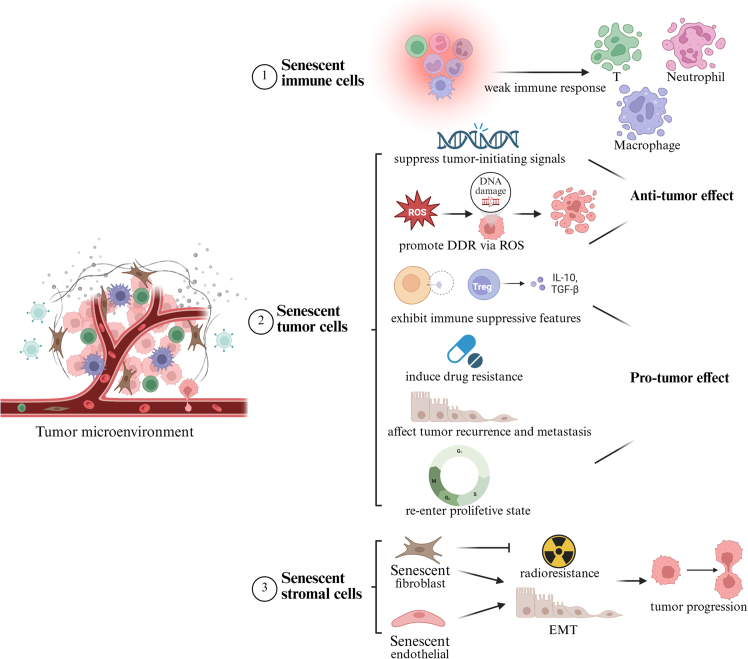
Influence of different components in tumor microenvironment upon tumor immune (created with BioRender.com)

The TME consists of tumor cells, immune cells, and stromal components such as fibroblasts and endothelial cells (ECs). These components exert distinct effects when they become senescent. STCs initially exhibits a protective role; however, as they accumulate, they release pro-inflammatory factors via SASP, thereby accelerating tumor progression. As the tumor advances, immune cell senescence exacerbates immune suppression. Meanwhile, senescent fibroblasts and ECs contribute to tumor metastasis and treatment resistance. The impact of various senescent cell types on tumor immunity is illustrated in Figure 3.

### Effect of STCs

Senescence may exert a paradoxical effect on tumor cells. It induces cell-cycle arrest and halts tumor cell proliferation. On the other hand, senescence promotes the growth and invasion of neighboring tumor cells through SASP, while also contributing to resistance against therapies.

#### CS initially exhibits anti-tumor effect

CS represents a critical tumor-suppressive mechanism that limits malignant transformation by halting cell proliferation. One of the key regulators, homeodomain-only protein X (HOPX), an atypical homeodomain protein, exerts its tumor-suppressive function by promoting CS through activation of the Ras/MAPK signaling pathway and concurrent inhibition of the AKT pathway in multiple NSCLC cell lines such as H1975 and A549.^62^ In contrast, MYC proto-oncogene (MYC) plays a pivotal oncogenic role in lung tumorigenesis by antagonizing BRAFV600E-induced senescence in mouse lung tumor model, thereby facilitating tumor progression and reducing survival.^63^ Supporting this, He et al. demonstrated that miR-34a enhances IR-induced senescence in human NSCLC cells by directly targeting MYC, leading to suppressed tumor cell proliferation.^64^ Senescence acts as a natural barrier in the early stages of tumorigenesis, largely through the activation of canonical tumor suppressor pathways. For example, CDC25B, a phosphatase regulating cell cycle progression, promotes p53 stabilization by dephosphorylation and thereby triggers senescence, demonstrated in human lung fibroblasts IMR90 and p53-wild-type H1299, ultimately constraining malignant transformation.^65^

In addition to oncogene signaling, ROS and DDR pathways also mediate senescence induction. Proline dehydrogenase (PRODH) suppresses proliferation and survival of lung adenocarcinoma (LUAD) cells (NCI-H1299) by promoting ROS accumulation and upregulating markers of senescence, including SA-β-gal, p21, and SASP factors.^66^ Similarly, inhibition of Hsp90 disrupts oncogenic RAF kinase, further induces DDR, and triggers CS, which serves as an effective tumor-suppressive mechanism and is associated with reduced metastatic lesions in the A549 human NSCLC xenograft mouse model.^67^ A membrane-stable mutant of CD40 ligand (CD40L-M) has been shown to induce senescence in CD40-positive NSCLC cell lines A549 and H460 through activation of the ATM/Chk2-mediated DDR pathway, with additional involvement of NF-κB signaling.^68^

Notably, senescence can be induced independent of oxidative stress. The recombinant fungal immunomodulatory protein reFIP-gts from *Ganoderma tsugae* suppresses telomerase activity and induces premature senescence in A549 lung cancer cells via p27-mediated CDK2 inhibition, independent of ROS.^69^ Moreover, depletion of CDCA3, a cell cycle regulatory protein, triggers senescence via p21 induction, independent of p53 or p16, and significantly inhibits tumor growth in multiple lung cancer models.^70^

#### Long-lived STCs drive tumor progression

While senescence initially impedes tumor progression by enforcing irreversible cell-cycle arrest, the persistence of STCs can foster an immunosuppressive TME and contribute to tumor progression through SASP.

Recent studies have demonstrated that tumors exhibiting elevated expression of senescence-associated signals such as *FOXM1*, *VDAC1*, *PPP3CA*, *MAPK13*, *PIK3CD*, *RRAS*, and *CCND3* exhibit distinct immune suppressive features and are negatively correlated with tumor-infiltrating neutrophils in LUAD transcriptomic cohorts (TCGA-LUAD plus multiple Gene Expression Omnibus (GEO) validation sets and an independent Chinese clinical cohort), with functional support *in vivo* from a Lewis lung carcinoma (LLC) mouse model showing neutrophil and CD8^+^ T cell modulation under FOXM1 inhibition.^71^ Loss of Calbindin, a calcium-binding protein, induces senescence in lung squamous cell carcinoma (LUSC) cells, which in turn promotes neutrophil infiltration through SASP-mediated CXCL8 secretion, ultimately correlating with worse prognosis. This was shown *in vitro* LUSC line models (HARA and LK-2), *in vivo* in xenografts, and in patient cohorts (TCGA LUSC cohort and TRACERx-100 multiregion biopsies).^72^ In the senescent TME, the expression of CD73 on macrophages is significantly upregulated, promoting the production of immunosuppressive adenosine, which significantly inhibits T cell proliferation and activity. This process is orchestrated by SASP cytokines secreted by STCs and the Janus kinase (JAK)/STAT3 signaling pathway in the LLC syngeneic model.^73^ Immunohistochemical analysis in the TCGA-LUSC cohorts reveals a significant downregulation of FOXP3^+^ regulatory T cells in the senescent microenvironment, indicating immune reprogramming.^74^

TIS has been implicated in drug resistance. In NSCLC cell line A549 and H1299, senescence-like tumor cells characterized by high *DNMT3A* expression exhibit resistance to apoptosis and contribute to tumor relapse following TKI therapy. Pemetrexed-resistant H460 cells also display a senescent phenotype, suggesting that TIS may underlie time-dependent chemoresistance.^75^

Senescent cells contribute significantly to tumor recurrence and metastasis. Despite cell-cycle arrest being a hallmark of senescence, certain senescent cells can escape this arrest and regain proliferative capacity. In NSCLC cell line H1299, this escape is licensed by common genomic contexts—particularly p53 loss with p16^INK4a^ deficiency, where aberrant *Cdk1* activity drives formation of polyploid senescent cells and enables depolyploidization, mediated cell-cycle re-entry; survivin suppresses apoptosis in this setting, while p27 restrains Cdk1 and limits escape.^76^ In a *KRAS*^*G12D*^ mouse model, Volonté et al. demonstrated that downregulation of endogenous Caveolin-1 bypasses oncogene-induced tumor cell senescence and promotes tumor initiation and progression.^77^ Additionally, some senescent cells can acquire stem-like properties, thus promoting recurrence. In lung cancer cell line H460, subtoxic doses of cisplatin induce senescence while upregulating stemness markers such as CD133 and CD44 via the GRP78/AKT signaling axis.^78^

SASP also facilitates extracellular matrix degradation and metastasis. In the 10Gy X-ray-induced senescent A549 cell line, the expression of E-cadherin decreased, while N-cadherin, vimentin, MMP9, and other markers were upregulated, demonstrating an upregulation of EMT in the cells.^79^ Liu et al. further confirmed that this IR-induced EMT shift is mediated by the TGF-β/Smad signaling: irradiation elevates TGF-β1 and *p*-Smad2/3 in A549 cell line.^80^

In summary, CS acts as an early brake on tumorigenesis but, if chronic, can drive progression through SASP-mediated remodeling, immune escape, stemness, and EMT. Recognizing these cell-source and time-dependent shifts, particularly in lung cancer, supports context-specific senescence-targeted strategies and rational combinations with immunotherapy.

### Effect of senescent immune cells

Senescent immune cells have distinct functional attributes that reshape immune responses and contribute to tumor progression and overall immune dysregulation.

Among immune subsets, T cells are particularly susceptible to senescence, which compromises their functionality within the TME. Senescent T cells in the TME, acquire dysfunctional mitochondria with mutated mtDNA transferred from cancer cells via tunneling nanotubes (TNTs) and EVs; this process triggers metabolic collapse, impairs effector functions and memory formation, and drives resistance to ICIs. This has been demonstrated *in vitro* using primary human tumor infiltrating lymphocytes (TILs) co-cultured with tumor lines (e.g., melanoma MEL04, breast MDA-MB-231) and *in vivo* in C57BL/6J syngeneic lung cancer (LLC/A11 and LLC/P29).^31^ In the study by Liu et al., they demonstrated that DNA damage and activation of the p38-MAPK pathway are key mediators of T cell senescence driven by murine regulatory T cells and tumor cells in LLC mouse model.^81^ Compared with the primary lesions of NSCLC, bone metastasis (BM) shows a higher infiltration of malignant cells with senescence characteristics and is involved in regulating T cell activation. Single-cell RNA sequencing of patient samples further indicates that BM-infiltrating T cells acquire a senescent immune phenotype: a decrease in naive T cells and an increase in CD4^+^Tstr cells. These senescent T cells are associated with enhanced immune suppression and pro-angiogenic activity.^82^

Senescent macrophages in the TME exhibit pro-tumor properties, and their selective clearance has been shown to restore anti-tumor immunity. Haston et al. utilized a novel p16-FDR transgenic mouse model to identify senescent macrophages in KRAS-driven lung cancer.^83^ Targeted depletion of these senescent cells in this lung cancer model led to a significant reduction in tumor burden and improved overall survival *in vivo*. Notably, macrophages with senescent features were detected in human lung pre-invasive lesions but were largely absent in established adenocarcinomas.^83^ Similarly, senescent alveolar macrophages exhibit pro-tumor characteristics, and their elimination facilitated cytotoxic T cell infiltration and delayed tumor initiation in *Kras* mice.^84^

Senescent programs in tumor-infiltrating neutrophils also foster lung-cancer progression. In human pulmonary adenocarcinoma, tumor-derived GM-CSF/G-CSF prolong the survival of alveolar neutrophils *in situ*, and neutralizing these cytokines reverses the anti-apoptotic activity, indicating a tumor-driven shift toward long-lived/aging-like neutrophils.^85^ Consistently, NSCLC single-cell maps reveal tissue-resident/maturation-skewed neutrophil states within tumors, supporting in-tumor reprogramming and extended persistence rather than the short-lived phenotype seen in homeostasis.^86^ Collectively, these data align with a model in which longer-lived, reprogrammed neutrophils in NSCLC foster immunosuppression and disease progression.

Senescent immune cells undergo functional reprogramming that actively promotes tumor progression and immunosuppression. Their accumulation within the TME weakens antitumor immune surveillance and promotes cancer development.

### Effect of senescent stromal components

Senescent stromal cells, including fibroblasts and ECs, promote tumor progression via sustained SASP and ECM remodeling. This section highlights their emerging roles in cancer biology.

Senescence in fibroblasts can be triggered by diverse intrinsic and extrinsic stimuli, which supports their pro-tumorigenic roles. Bischof et al. examined the biological properties of the promyelocytic leukemia gene (PML) in senescence assays using WI38 primary human fibroblasts. They demonstrated that PML overexpression triggers fibroblast senescence.^87^ IR has also been shown to induce premature senescence in CCD19Lu human lung fibroblasts by promoting ROS production and DDR. Additionally, IR activates p38-MAPK, a known inducer of premature senescence.^88^ Loss of mouse *TARSH (Abi3bp)* triggers p53-and p21^cip1^-dependent senescence in mouse fibroblasts.^89^ Acrolein, a toxic aldehyde component of cigarette smoke, accelerates senescence in IMR-90 human fibroblasts by inducing oxidative stress and mitochondrial dysfunction.^90^

Senescent fibroblasts act as potent drivers of tumor growth and metastasis primarily via SASP secretion. In the TME, senescent fibroblasts with elevated *DPP4* expression promote the proliferation of LUAD cell lines through IL-6 and IL-8 signaling. Likewise, radiation-induced senescent WI-38 fibroblasts also enhance the clonal growth of A549 cells, illustrating the pro-tumorigenic role of fibroblast-derived SASP.^91^

Senescent fibroblasts also contribute to therapy resistance. For instance, senescent fibroblasts promote the proliferation and inhibit apoptosis of human NSCLC cell lines, NCI-H292, A549, HCC827, and induce radioresistance through the IL-6/JAK/STAT3 signaling pathway.^26^ The chronic release of inflammatory mediators like IL-6, IL-1β and other SASP factors leads to aberrant immune cell infiltration, reshaping the TME. Pharmacologic or genetic blockade of key SASP axes (for example, IL-6/JAK-STAT3 inhibition, MMP blockade) or elimination of senescent fibroblasts with senolytics reverses these effects in preclinical models.^92^

Senescent ECs can affect tumor progression. In KRAS-driven NSCLC, p16^high^ senescent cells accumulate within the TME, with ECs among the predominant senescent populations; genetic senescent-cell clearance (or depletion of senescent macrophages) reduces tumor burden, implicating endothelial-cell senescence in tumor initiation and progression.^83^

In summary, senescent fibroblasts and ECs are critical components of the TME, contributing to tumor progression, metastasis, and treatment resistance. Their sustained SASP secretion and stromal remodeling capabilities reprogram the microenvironment toward a tumor-supportive state, making them promising therapeutic targets.

### Senescence-driven signaling networks

In lung cancer, senescence drives tumor progression through convergent signaling modules. STCs secrete SASP factors such as CXCL8/IL-8 and CCL2 that recruit and reprogram myeloid populations (e.g., MDSCs), establishing an immunosuppressive niche that supports invasion and metastatic spread. Therapy-induced senescent cells further upregulate PD-L1, DKK3, and CD73-adenosine/JAK-STAT3 signaling, directly suppressing T cell and NK surveillance. Persistent senescent clones can aberrantly activate Cdk1/survivin in p53/p16-deficient NSCLC, escape arrest, re-enter the cell cycle, and seed relapse. In parallel, senescent immune cells (senescent T cells, macrophages, and long-lived neutrophils) adopt dysfunctional, pro-tumor states that sustain immunosuppression and blunt cytotoxic effector function. Senescent stromal cells, particularly irradiation-induced fibroblasts and endothelial-like cells, provide IL-6/JAK-STAT3-, TGF-β/EMT-, and matrix-remodeling signals that drive proliferation, survival, treatment resistance, angiogenic support, and dissemination. Together, these circuits mechanistically link senescence to immune escape, EMT-driven invasion and metastasis, therapy tolerance, and recurrence in NSCLC.

## Multifaceted application of CS in the treatment of lung cancer

CS, characterized by an irreversible arrest of the cell cycle, exerts a dual role in cancer biology initially functioning as a tumor-suppressive mechanism, while paradoxically promoting tumor progression in its persistent state. This complex nature of senescence presents multiple therapeutic opportunities in lung cancer, ranging from strategies that induce senescence to halt tumor growth, to approaches that selectively eliminate senescent cells (senolytics) or modulate their secretory profile (senomorphics).

### Utilization of CS as a therapeutic modality in lung cancer

Inducing CS in tumor cells has emerged as a promising anticancer strategy. Many conventional and novel agents exert their effects by promoting senescence through mechanisms involving DNA damage, oxidative stress, telomere attrition, or cell cycle dysregulation.

#### TIS in lung cancer

For instance, resveratrol (RSV) leads to the upregulation of *Rad9* and *p21* and the establishment of a senescent state in A549 and H460 cells.^93^ Klotho, a well-known anti-aging protein, inhibits lung cancer initiation and progression by modulating the SASP in A549 cells.^94^ Pterostilbene (PT), a dimethylated analog of RSV, induces senescence by downregulating *hTERT* and telomerase activity in a p53-dependent manner, thereby initiating DDR and cell-cycle arrest across NSCLC cell lines H460 and H1299.^95^ Extracellular adenosine likewise induces durable p53/p21-dependent senescence in A549 cells and suppresses tumor growth in xenografts, with persistent γH2AX and pCHK2 signaling marking a prolonged DDR.^96^ Additional compounds such as volasertib, a polo-like kinase 1 (Plk1) inhibitor, induces senescence across A549 and NCI-H1975, partially through p53-mediated mechanisms.^97^ The ginger extract ZOE, appears to similarly inhibit *hTERT* expression and telomerase activity in A549 lung cancer cells.^98^ PIP, a platinum (II) phenanthroimidazole G-quadruplex DNA stabilizing ligand, selectively induces telomere erosion and senescence in telomerase-positive A549 cells while sparing normal cells.^99^ Artemether upregulates *p16* expression, and is a promising novel candidate drug in NSCLC, capable of inducing p53-independent senescence, apoptosis, and growth arrest across A549 and NCI-H1299.^100^

Senescence induction also interacts with immune modulation, impacting the effectiveness of immunotherapies. Tumors with lower levels of senescence show greater immune infiltration and upregulated immune checkpoint activity both *in vitro* and *in vivo*. SASP factors such as TNF-α and ICAM-1 enhance NK cell surveillance.^33^ In a melanoma model, blockade of DNA damage or p38 signaling to prevent T cell senescence, in combination with anti-PD-L1 treatment, can synergistically enhance the anti-tumor efficacy of adoptive T cell transfer therapy.^81^ Increasing PD-L1 expression through TIS can benefit patients with low PD-1/PD-L1 expression from anti-PD-1/PD-L1 therapy.^101^ Together these observations support pairing senescence induction with immune checkpoint therapy while monitoring SASP programs that might tip toward immune escape.

Beyond immunotherapy, senescence induction can enhance sensitivity to radiotherapy and chemotherapy. Marchantin C (Mar-C) combined with sulfated curdlan induces tumor senescence with stronger anti-tumor effects than doxorubicin (DOX).^102^ Knockdown of the E3 ligase C-terminus of Hsc70-interacting protein (CHIP) reduces the ubiquitination and degradation of *p21*, thereby enhancing the sensitivity of lung cancer cells to IR through p21-mediated senescence.^103^ In EGFR-addicted and non-addicted NSCLC models, combining EGFR inhibition with IR increases DNA double-strand breaks (DSBs) persistence, disrupts MEK-ERK signaling, and provokes pronounced senescence rather than apoptosis, translating into robust radiosensitization.^104^

Although senescence induction can suppress tumor growth, persistent senescent cells may create a pro-tumorigenic environment via chronic SASP secretion, promoting immune evasion, EMT, and recurrence. To address this paradox, clinical and preclinical senotherapeutics have been developed to either eliminate or reprogram senescent cells. They are differentiated into senolytics (clearing senescent cells) and senomorphics (modulating senescence-associated phenotypes; sometimes termed senostatics when primarily blunting senescence maintenance or SASP without killing cells). Representative agents are summarized in Table 2.

**Table 2:** Senotherapeutics in cancer: Senolytics, senomorphics, and senostatics

Class	Mechanism or type	Agent(s)	Evidence	Primary action
Senolytic	BCL-2/BCL-xL inhibitor	navitoclax (ABT-263)	clinical trial (early-phase; NSCLC/SCLC; combos)	senescent-cell apoptosis via BCL-2 family blockade
Senolytic	β-gal-activated prodrug	Nav-Gal (galacto-conjugated navitoclax)	preclinical (NSCLC A549; *in vivo*)	selective senolysis of TIS cells
Senolytic	BCL-xL degrader (PROTAC)	DT2216	clinical trial (FIH, solid tumors incl. lung)	platelet-sparing senolysis
Senolytic	natural product (multi-target)	fisetin	preclinical (NSCLC/SCLC)	senolysis; reverses TIS-linked resistance
Senolytic	SRC/tyrosine kinase inhibitor	dasatinib + quercetin	preclinical; limited lung-specific	target SCAP/TK pathways
Senolytic	p53/FOXO axis	FOXO4-DRI	preclinical	disrupt p53-FOXO to kill senescent cells
Senolytic	p53/MDM2 axis	RG7112	preclinical/early clinical trial	inhibit MDM2 (stabilize p53)
Senolytic	HSP90 inhibitor	alvespimycin (17-DMAG)	preclinical	destabilize HSP90 clients; senolysis
Senolytic	BCL-2 family inhibitor	ABT-737	preclinical	BCL-2 inhibition
Senolytic	cardiac glycosides	digoxin	preclinical	Na^+^/K^+^-ATPase targeting; senolysis
Senolytic	cardiac glycosides	ouabain	preclinical	Na^+^/K^+^-ATPase targeting; senolysis
Senolytic	natural product (multi-target)	piperlongumine	preclinical	PI3K/AKT; BCL-2-linked senolysis
Senolytic	natural product (multi-target)	procyanidin C1	preclinical	BCL-2 family targeting
Senolytic (other)	epigenetic	ARV825 (BRD4 degrader)	preclinical	BRD4-dependent senolysis
Senolytic (other)	immune-mediated clearance	CAR-T cells	concept-stage	immune clearance of senescent cells
Senomorphic	p38-MAPK inhibition	SB203580	preclinical (lung fibroblasts → NSCLC)	SASP down-tuning (IL-6 dominant); EMT/invasion ↓
Senomorphic	JAK/STAT inhibition	ruxolitinib	clinical exploration (NSCLC combos); pulmonary models	SASP attenuation (IL-6-STAT3)
Senomorphic	NF-κB/SASP modulators	hydroxytyrosol	preclinical	lower IL-6/IL-8/MMP SASP
Senomorphic	NF-κB/SASP modulators	oleuropein	preclinical	lower IL-6/IL-8/MCP-1/RANTES
Senomorphic	NF-κB/SASP modulators	apigenin	preclinical	SASP modulation
Senomorphic	p38-MAPK inhibitors (others)	UR-13756; BIRB-796	preclinical	SASP hub kinase inhibition
Senomorphic/senostatic (metabolic)	mevalonate pathway/YAP-TAZ down-tuning	simvastatin	clinical exploration (NSCLC with EGFR-TKI); lung stroma models	blunt SASP; reduce EMT/invasion signaling
Senomorphic/senostatic	DDR→ATM→NF-κB/SASP axis	KU-55933 (ATM inhibitor)	preclinical (A549; RT/chemo-induced models)	suppress senescence maintenance & SASP
Senostatic	AMPK-mTOR axis	metformin	preclinical/translational (lung TME relevant)	reprogram SASP; blunt IL-6-STAT3
Senostatic	mTOR inhibitor	rapamycin	preclinical	restrain SASP maintenance

#### Senolytics (clearing senescent cells)

Senolytics selectively eliminate senescent cells and mitigate their harmful effects. Among senolytics, the BCL-2/BCL-xL inhibitor navitoclax has generated the most lung cancer-specific clinical data. In EGFR-mutant NSCLC, a phase 1b study of osimertinib plus navitoclax established a recommended phase-2 dose and showed manageable hematologic toxicity, supporting the feasibility of a TIS-to-senolysis sequence in patients (ETCTN 9903).^105^ In SCLC, early trials of single-agent navitoclax documented on-target thrombocytopenia as the dose-limiting toxicity but also signs of activity and biomarker correlations, informing subsequent combination strategies.^106^^,^^107^ To mitigate platelet toxicity while preserving senolysis, BCL-xL degraders (PROTACs) have entered translation; the first-in-human study of DT2216 is ongoing in advanced solid tumors, outlining a platelet-sparing senolytic path for lung cancers (NCT06620302). A complementary preclinical direction is senolytic pro-drugging that exploits senescence-associated β-galactosidase. Nav-Gal, a galacto-conjugated navitoclax, selectively clears cisplatin-induced senescent A549 cells and eradicates STCs *in vivo* while reducing normal-tissue cytotoxicity, thereby enhancing chemotherapy efficacy.^108^ In the genetic niche, fisetin reverses *EIF3A* R803K-linked chemotherapy resistance by targeting the senescent phenotype in SCLC models, hinting at genotype-guided senolysis.^109^

#### Senomorphics/senostatics (reprogramming SASP and phenotypes)

Senomorphics focus on altering the phenotypes of senescent cells to prevent their detrimental effects, such as SASP secretion, while preserving their tumor-suppressive properties. These agents target specific pathways to modulate the impact of senescence on the TME and cancer therapy. p38-MAPK activity is required for SASP induction across senescent fibroblast models; pharmacologic inhibition (SB203580) suppresses IL-6-dominant SASP programs that are known to fuel EMT and invasion.^110^ Beyond p38, JAK/STAT blockade (ruxolitinib) functions as a senomorphic by attenuating SASP; while clinical SASP-targeting data in NSCLC are still limited, ruxolitinib has shown senomorphic activity in pulmonary disease models and has been clinically explored with platinum/pemetrexed backbones in NSCLC, supporting translational plausibility.^111^ Metformin, a metabolic senostatic, can reprogram SASP and blunt IL-6-STAT3 signaling though most tumor-type data are preclinical or from non-lung settings.^112^

### Prediction of survival and treatment response related to senescence

Senescence-related scoring models provide valuable insights into the immunosuppressive landscape of tumors and their implications for prognosis and treatment response. Lin et al. constructed a novel survival prediction model-CS score (CSS) based on the expression of four cell senescence cluster-related differentially expressed genes (*C1QTNF6*, *SQOR*, *FAM83B*, and *LYPD3*). Patients with low CSS exhibited significantly prolonged survival, whereas the high CSS group was characterized by elevated infiltration of immunosuppressive cells, including MDSCs and Tregs. Additionally, the expression of PD-L1 and co-inhibitory immune checkpoint molecules such as TIM3 and LAG3 were significantly upregulated in the high CSS group. These suggest that while senescence contributes to immune suppression, CSS may serve as a predictive biomarker for immunotherapy responsiveness.^113^ Conversely, low-risk or low-senescence groups tend to show enhanced immune cell infiltration and upregulation of immune checkpoint-related genes, supporting their potential responsiveness to ICIs.^114^ Moreover, tumors with elevated expression of senescence-associated genes such as *FOXM1*, *VDAC1*, *PPP3CA*, *MAPK13*, *PIK3CD*, *RRAS*, and *CCND3* exhibit distinct immunosuppressive features and are inversely correlated with neutrophil infiltration in the TME, further reinforcing their prognostic and predictive value, particularly in LUAD.^71^ Senescence-associated gene signatures—particularly mRNA-based markers—offer a promising approach to assess prognosis and forecast the efficacy of immunotherapy, especially in patients undergoing ICI-based regimens.

Beyond their prognostic utility, senescence-associated mechanisms offer multifaceted therapeutic opportunities in cancer. CS exhibits dual effect toward anti-tumor therapy. To address this paradox, an integrated therapeutic strategy combining TIS with senotherapeutics can optimize tumor suppression. These include leveraging senolytics to clear harmful senescent cells and senomorphics to reprogram their phenotypes. These strategies hold immense potential to enhance therapeutic efficacy, minimize recurrence, and pave the way for innovative, personalized cancer treatments.

## Discussion

CS is increasingly recognized as a complex biological process that exerts both tumor-suppressive and tumor-promoting effects. In lung cancer, TIS has emerged as a potential mechanism to halt tumor progression by arresting cell proliferation and enhancing responsiveness to radiotherapy and immunotherapy. However, persistent senescent cells in the TME can secrete pro-inflammatory SASP factors, promote EMT, and facilitate immune evasion, ultimately contributing to therapeutic resistance and disease recurrence.

These dual effects of senescence underscore the need for refined clinical strategies. Recent advances in senotherapeutics, including senolytics and senomorphics offer promising avenues to enhance the therapeutic benefits of TIS while minimizing its adverse consequences. Preclinical studies suggest that combining senescence-inducing therapies with senotherapeutics may represent a more effective and durable treatment model in lung cancer.

From a precision medicine perspective, senescence-related gene signatures and scoring systems, such as CSS, have demonstrated value in predicting prognosis and immunotherapy response. These tools may help stratify patients and guide selection of immunotherapy or combination regimens. However, their translation into clinical practice requires further validation across independent cohorts and platforms.

Senescence-targeted approaches are emerging as a promising component of multimodal treatment strategies in lung cancer. By inducing senescence to suppress tumor growth and subsequently modulating or eliminating senescent cells, these strategies offer the potential to enhance therapeutic efficacy, reduce recurrence, and improve long-term disease control. When carefully implemented, this dual approach may help overcome limitations associated with traditional therapies and support the advancement of personalized cancer care.

However, several key challenges must be addressed before senescence-based therapies can be widely adopted in clinical practice. First, there is a lack of reliable and non-invasive biomarkers capable of dynamically monitoring senescence status and treatment response, which limits the ability to guide and adapt therapeutic decisions. Second, the optimal timing and duration for either inducing or clearing senescent cells remain unclear, raising concerns about tumor adaptation and potential harm to normal tissues. Third, the risk of off-target effects associated with senotherapeutic agents underscores the need for tumor-specific delivery systems and further safety optimization. Continued research is essential to address these barriers and fully realize the clinical utility of senescence-directed treatment strategies in lung cancer.

## Conclusion

The TME in lung cancer harbors a variety of senescent cell types, including not only tumor cells but also immune cells, fibroblasts, and ECs. These senescent cells contribute to disease progression through persistent secretion of SASP factors, impairment of immune surveillance, remodeling of the extracellular matrix, and promotion of angiogenesis and metastasis. Collectively, these alterations establish a pro-tumorigenic niche that facilitates cancer growth and undermines treatment efficacy.

To counteract these effects, senescence-targeted therapies have emerged as promising strategies. Senotherapeutics have shown potential in preclinical studies. By modulating the senescent cell burden within the microenvironment, these therapies may enhance antitumor immunity, restore treatment sensitivity, and improve overall therapeutic outcomes.

Moving forward, effective clinical translation will require the development of reliable, cell type-specific senescence markers, optimized drug delivery systems, and clear guidance on the timing of intervention. Integrating these approaches into existing lung cancer treatment regimens could disrupt the supportive role of senescent cells in the TME and provide new avenues for durable disease control.

## Acknowledgments

We thank colleagues in our teams for insightful discussions. We also acknowledge the authors of the studies cited in this review for advancing the field. Support for this work is provided by 10.13039/100018904Beijing Xisike Clinical Oncology Research Foundation, China (Y-2023AZMETQN-0066), 10.13039/501100003819Natural Science Foundation of Hubei Province, China (2025AFD835), and 10.13039/100014718National Natural Science Foundation of China (NSFC), China (82472969).

## Author contributions

Z.X., writing – original draft – review and editing; F.L., review and editing; Q.C. and S.H., funding acquisition, review, and editing. All authors contributed to this manuscript. All authors read and approved the final.

## Declaration of interests

No competing interests were declared.
